# Supplementary material for: Comparative Analysis of Hydrophilic Ingredients in Toad Skin and Toad Venom Using the UHPLC-HR-MS/MS and UPLC-QqQ-MS/MS Methods Together with the Anti-Inflammatory Evaluation of Indolealkylamines
Source: Molecules. 2018 Dec 27;24(1):86. doi: 10.3390/molecules24010086 (PMC6337282; doi:10.3390/molecules24010086)
Supplement: Supplementary file 1 [file molecules-24-00086-s001.pdf]

Comparative Analysis of Hydrophilic Ingredients in Toad Skin and Toad Venom Using the UHPLC-HR-MS/MS and UPLC-QqQ-MS/MS Methods Together with the Anti-Inflammatory Evaluation of Indolealkylamines

Yu Zhang [1], Bo Yuan [2], Norio Takagi [2], Hongjie Wang [1], Yanyan Zhou [1], Nan Si [1], Jian Yang [1], Xiaolu Wei [1], Haiyu Zhao [1,*] and Baolin Bian [1,*]

[1] Institute of Chinese Materia Medica, China Academy of Chinese Medical Sciences, Beijing 100700, China; zhangyuddnh@163.com (Y.Z.); hjwang@icmm.ac.cn (H.W.) yyzhou@icmm.ac.cn (Y.Z.); nsi@icmm.ac.cn (N.S.); jyang@icmm.ac.cn (J.Y.); xlwei@icmm.ac.cn (X.W.)

[2] Department of Applied Biochemistry, Tokyo University of Pharmacy & Life Sciences, 1432-1 Horinouchi, Hachioji, Tokyo 192-0392, Japan; yuanbo@toyaku.ac.jp (B.Y.); takagino@toyaku.ac.jp (N.T.)

* Correspondence: hyzhao@icmm.ac.cn (H.Z.); blbian@icmm.ac.cn (B.B.); Tel.:+136-610-784-32(H.Z.); +139-012-278-22 (B.B.)

Table S1. Parameters of UPLC-QqQ-MS/MS analysis for eleven compounds

Compounds	Rt /min	MRM				
		Q1/Q3 ion[a] (*m/z*)	DP/V	CE/eV	CXP/V	EP/V
Valine	1.08	118.0/72.1	30	11	11	10
Adenine	1.39	136.1/119.2	34	31	16	10
Nicotinic acid	1.68	124.0/80.0	102	31	13	10
Hypoxanthine	2.10	137.2/120.1	100	32	7	10
Xanthine	2.68	153.0/136.2	149	25	15	10
Serotonin	4.44	177.1/160.2	92	14	10	10
N-methyl serotonin	4.95	191.0/160.1	45	15	11	10
Bufotenidine	5.35	219.2/160.1	42	23	25	10
Bufotenine	5.72	205.1/160.1	24	24	14	10
Dehydrobufotenine	7.30	203.0/188.1	102	31	17	10
Bufothionine	7.72	283.0/203.2	130	21	16	10
Diazepam	8.72	284.9/193.1	167	43	12	10
Ion spray voltage (IS) /V			5500			
Ion source (GS1) setting/Psi			55			
Ion source (GS2) setting/Psi			55			

[a] Q1: precursor ion selected in Q1; Q3: product ion selected in Q3;.

Table S2. The regression equation, LOD and LOQ of the 11 standards in toad skin samples

Analyte	Regression equations	*R*	Linear range (μg/mL)	LOD (ng/mL)	LOQ (ng/mL)	Repeatability (n=6)	Stability (RSD%)	Intra-day precision (RSD%, n=6)	Inter-day precision (RSD%, n=3)
Valine	$Y=3.8355\times10^{6}X+2.4571\times10^{6}$	0.9997	0.21-43.48	0.0095	0.047	2.69	2.05	2.15	2.33
Adenine	$Y=1.0274\times10^{7}X+1080200$	0.9997	0.15-6.05	0.33	3.30	0.71	2.95	1.15	1.95
Nicotinic acid	$Y= 881906X+2016.4$	0.9995	0.11-4.60	2.30	11.50	2.37	1.24	1.50	1.84
Hypoxanthine	$Y=2.200\times10^{6}X+3688.9$	0.9995	0.23-93.75	2.34	11.72	2.93	2.69	2.09	2.39
Xanthine	$Y= 4400200X+2335.1$	0.9994	0.42-42.25	0.99	4.95	3.21	3.35	2.64	3.05
Serotonin	$Y=1.1181\times10^{6}X+1.5718\times10^{6}$	0.9988	2.38-38.20	24.76	49.52	1.37	2.24	0.25	3.38
N-methyl serotonin	$Y= 1.97615\times10^{7}X+713317$	0.9999	3.00-48.00	7.74	38.71	2.44	3.31	1.59	3.01
Bufotenidine	$Y= 6.5895\times10^{7}X+387000$	0.9995	23.37-374.00	1.30	6.50	1.36	2.34	2.26	2.76
Bufotenine	$Y= 4.1411\times10^{6}X+1395.8$	0.9989	12.93-414.00	25.88	103.52	2.90	3.46	1.30	3.19

Dehydrobufotenine	$Y= 3.2817\times10^{8}X+154440$	0.9991	3.00-48.00	0.0069	0.069	2.56	3.38	1.26	1.68
Bufothionine	$Y=4.3126\times10^{7}X+1.1772\times10^{5}$	0.9995	3.07-153.75	0.77	3.84	2.08	2.29	0.66	1.49

Table S3. The regression equation, LOD and LOQ of the eleven standards in toad venom samples

Analyte	Regression equations	*R*	Linear range (μg/mL)	LOD (ng/mL)	LOQ (ng/mL)	Repeatability (n=6)	Stability (RSD%)	Intra-day precision (RSD%, n=6)	Inter-day precision (RSD%, n=3)
Valine	Y=5.3156×$10^6$$X$+1.1318×$10^6$	0.9997	0.043-1.08	0.0095	0.047	1.04	2.85	2.15	2.33
Adenine	Y=1.0274×$10^7$$X$+1080200	0.9997	0.15-6.05	0.33	3.30	1.87	3.31	1.15	1.95
Nicotinic acid	Y=5.1613×$10^6$$X$+8.2313×$10^4$	0.9995	0.023-1.15	2.30	11.50	2.69	2.72	1.50	1.84
Hypoxanthine	Y= 818537X+167954	0.9985	0.023-3.75	2.34	11.72	2.20	2.49	2.09	2.39
Xanthine	Y= 4129000 X+3264.3	0.9999	0.016-8.45	0.99	4.95	2.34	1.62	2.64	3.05
Serotonin	Y=1.3172×$10^6$$X$+2.6083×$10^5$	0.9999	0.11-4.77	24.76	49.52	1.32	2.72	0.25	3.38
N -methyl serotonin	Y=1.9476×$10^7$$X$+13650	0.9999	30.00-1920.00	7.74	38.71	1.98	2.00	1.59	3.01
Bufotenidine	Y=6.5895 ×$10^7$$X$+387000	0.9995	233.70–3740.00	1.30	6.50	2.10	1.25	2.26	2.76
Bufotenine	Y=4.1411×$10^6$$X$+1395.8	0.9989	129.37-1035.00	25.88	103.52	2.93	2.45	1.30	3.19

Dehydrobufotenine	$Y=4.7837\times10^{6}X-12281$	0.9995	60.00-480.00	0.0069	0.069	2.34	2.87	1.26	1.68
Bufothionine	$Y=3.3481\times10^{7}X+1.0016\times10^{6}$	0.9998	0.03-3.07	0.77	3.84	0.36	2.77	0.66	1.49

Table S4. Recovery of eleven compounds in toad skin (*n*=3)

Analyte	Initial amount (μg)	Added amount (μg)	Total recovered amount(μg)	Recovery (%)	RSD (%)
Valine	19.9426	15.9859	36.1297	101.2583±3.0115	2.97
	19.9028	19.9824	40.2451	101.8012±0.5798	0.56
	19.9824	23.9789	43.5476	98.2748±2.1667	2.20
Adenine	1.2409	0.9969	2.2281	99.0232±1.2490	1.26
	1.2384	1.2461	2.4996	101.2111±2.7558	2.72
	1.2434	1.4953	2.7646	101.7285±2.0047	1.97
Nicotinic acid	3.1768	2.5219	5.7724	102.9229±1.6638	1.61
	3.1704	3.1524	6.3636	101.2923±1.9745	1.94
	3.1831	3.7829	6.8594	97.1825±1.0219	1.05
Hypoxanthine	17.3134	13.9372	30.9724	99.3258±1.7687	1.78
	17.2788	17.4215	34.8688	96.6490±0.6166	0.63
	17.3479	20.9058	38.2977	102.3880±2.0592	2.01
Xanthine	12.2107	9.7004	21.8125	103.0415±0.3191	0.31
	12.1863	12.1255	24.3112	103.2720±1.2903	1.24
	12.2350	14.5506	26.7197	101.7960±1.1414	1.12
Serotonin	12.4092	9.8867	22.3880	100.9306±2.6421	2.61
	12.3845	12.3584	25.0499	102.4840±1.3794	1.34
	12.4340	14.8301	27.4797	101.4540±3.1334	3.08
N -methyl serotonin	10.2749	8.3142	18.3882	97.5843±1.4335	1.46
	10.2544	10.3927	20.9582	102.9942±0.3299	0.29
	10.2953	12.4712	23.0443	102.2271±0.4390	0.42
Bufotenidine	102.2819	81.0846	186.4367	103.7865±0.8486	0.82

	102.0779	101.3557	202.7663	99.3417±1.2894	1.29
	102.4859	121.6268	226.1888	101.7068±0.8010	0.78
Bufotenine	47.5919	38.4235	87.7697	104.5655±0.3481	0.33
	47.4970	48.0294	95.6302	100.2161±0.7315	0.73
	47.6869	57.6353	104.3142	98.2512±1.3342	1.35
Dehydrobufotenine	22.6642	16.5515	38.9057	98.1267±2.0924	2.13
	22.6190	20.6894	43.3865	100.3772±1.7402	1.73
	22.7094	24.8273	48.4912	103.8443±1.5376	1.48
Bufothionine	54.3128	44.0750	99.4469	102.4029±2.6966	2.63
	54.2044	55.0938	107.1919	96.1876±0.0893	0.09
	54.4211	66.1126	118.4191	96.8016±0.6902	0.71

Table S5. Recovery of eleven compounds in toad venom (*n*=3)

Analyte	Initial amount (μg)	Added amount (μg)	Total recovered amount(μg)	Recovery (%)	RSD (%)
Valine	0.2507	0.2010	0.4457	97.0180±1.6119	1.66
	0.2467	0.2513	0.5000	100.7959±2.8695	2.84
	0.2508	0.3016	0.5499	99.1732±3.0881	3.11
Adenine	4.0673	3.3012	7.3935	100.7571±2.5815	2.56
	4.0131	4.1265	8.2363	102.3448±1.7186	1.67
	4.0809	4.9518	9.1164	101.6906±2.2498	2.21
Nicotinic acid	0.2212	0.1751	0.3905	96.7029±1.2627	1.30
	0.2182	0.2189	0.4314	97.3785±1.0159	1.04
	0.2219	0.2627	0.4735	95.8006±1.2395	1.29
Hypoxanthine	0.2212	0.1751	0.3905	99.9552±3.3.2176	3.21
	0.2182	0.2189	0.4314	101.7055±0.9623	0.94
	0.2219	0.2627	0.4735	102.5305±2.9664	2.89
Xanthine	1.7115	1.3219	3.0640	98.5530±2.8588	2.90
	1.6887	1.6524	3.3693	99.4378±2.2129	2.22
	1.7172	1.9829	3.7503	97.1840±2.0844	2.14
Serotonin	0.8173	0.6283	1.4461	100.0699±1.7300	1.72
	0.8064	0.7854	1.5732	97.6347±2.1333	2.18
	0.8200	0.9425	1.7311	96.6671±2.3877	2.47
N -methyl serotonin	141.5192	111.3772	252.0478	99.2381±0.8129	0.82
	139.6323	139.2215	282.2270	102.4229±1.0194	0.99
	141.9910	167.0658	308.9295	99.9238±2.2751	2.27
Bufotenidine	1292.2692	1030.5726	2330.4032	100.7337±3.0255	3.00

	1275.0390	1288.2158	2553.2371	99.2224±1.3035	1.31
	1296.5768	1545.859	2848.4041	100.3861±1.0842	1.08
Bufotenine	495.9808	384.7958	881.5789	100.2085±1.0827	1.08
	489.3677	480.9947	968.6293	99.6397±2.9791	2.98
	497.6340	577.1936	1064.7046	98.2462±1.1273	1.14
Dehydrobufotenine	237.5481	184.3548	425.5484	101.9774±1.2692	1.24
	234.3808	230.4435	466.2928	100.6373±1.5951	1.58
	238.3399	276.5322	513.0391	99.3372±0.7294	0.73
Bufothionine	1.9904	1.5723	3.5007	96.0586±2.4862	2.58
	1.9638	1.9654	3.9211	99.5872±2.7544	2.76
	1.9970	2.3585	4.3017	97.7189±1.2760	1.30

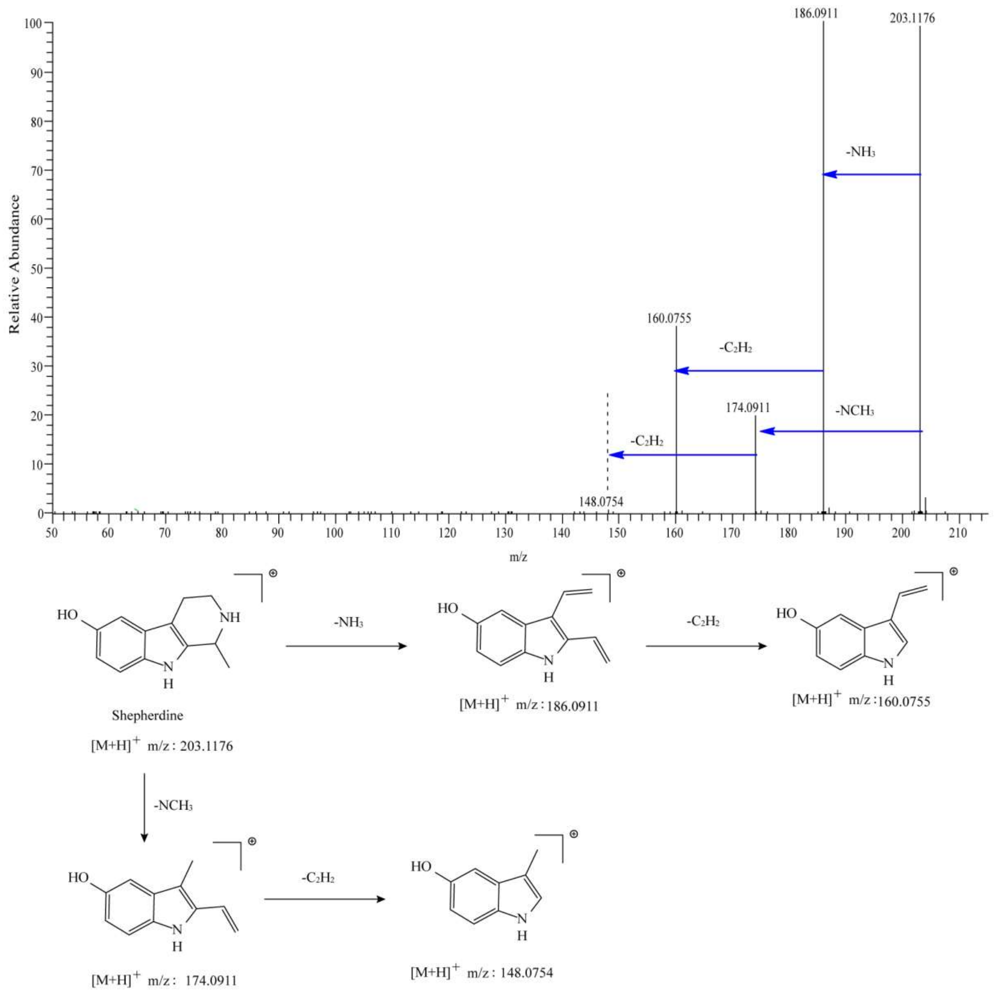

Figure S1. The MS/MS spectrum and fragmentation patterns of shepherdine

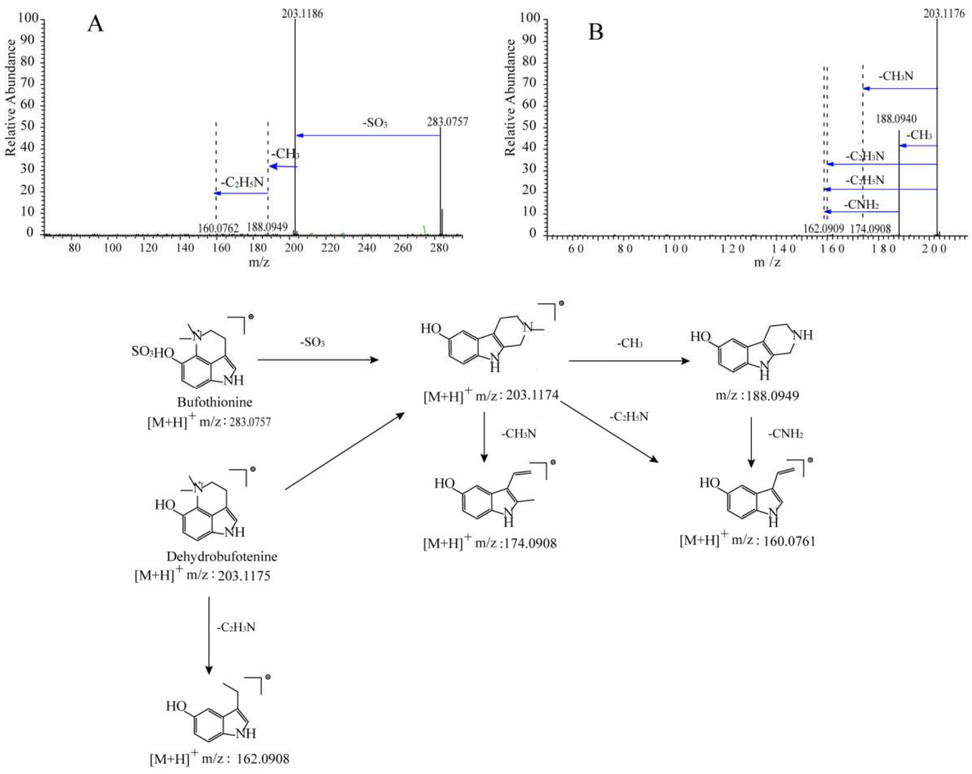

Figure S2. The MS/MS spectrum and fragmentation patterns of A: bufothionine; B: dehydrobufotenin

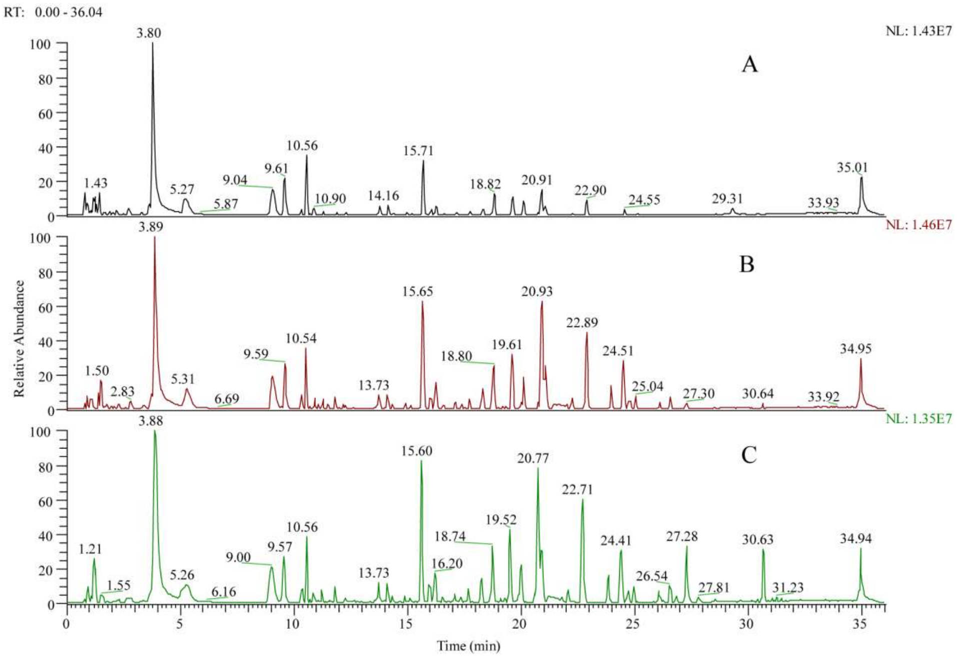

Figure S3. Optimized extraction conditions selected from three extraction solvents. A: water; B: 50% methanol; C: 50% ethanol.

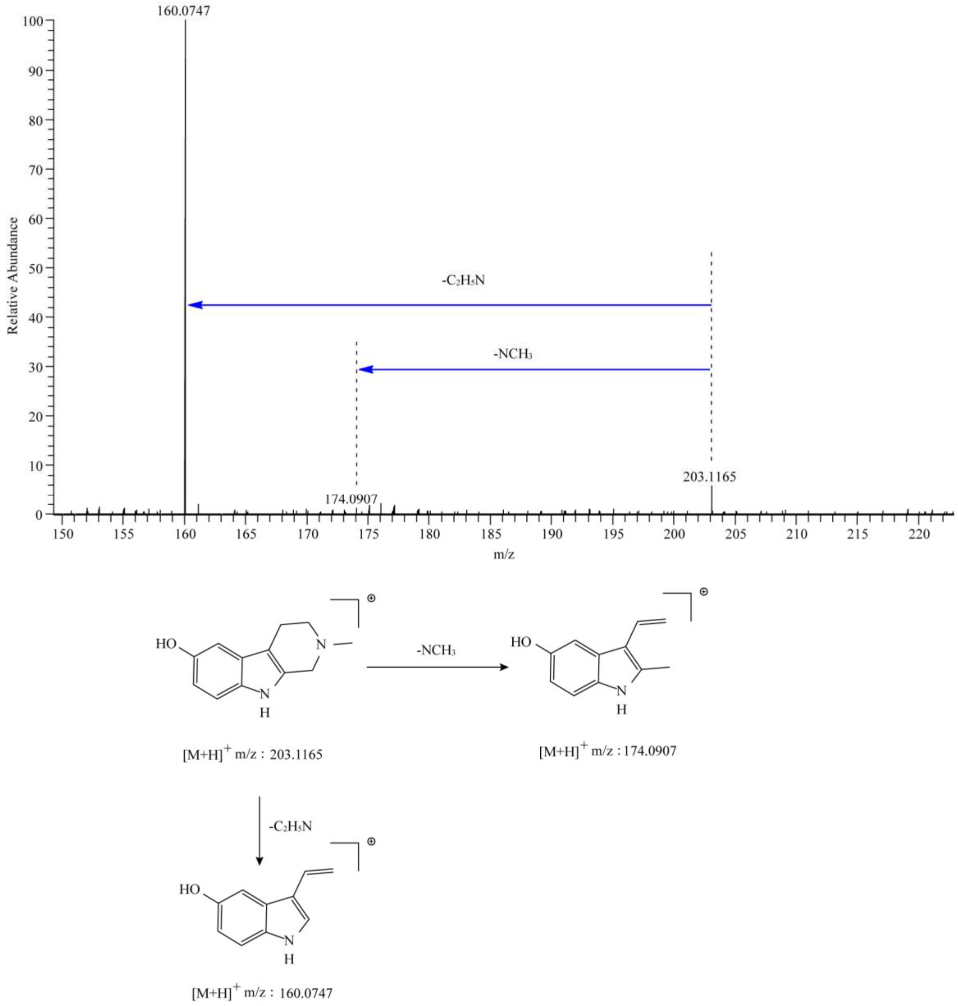

Figure S4. The MS/MS spectrum and fragmentation patterns of peak **19**

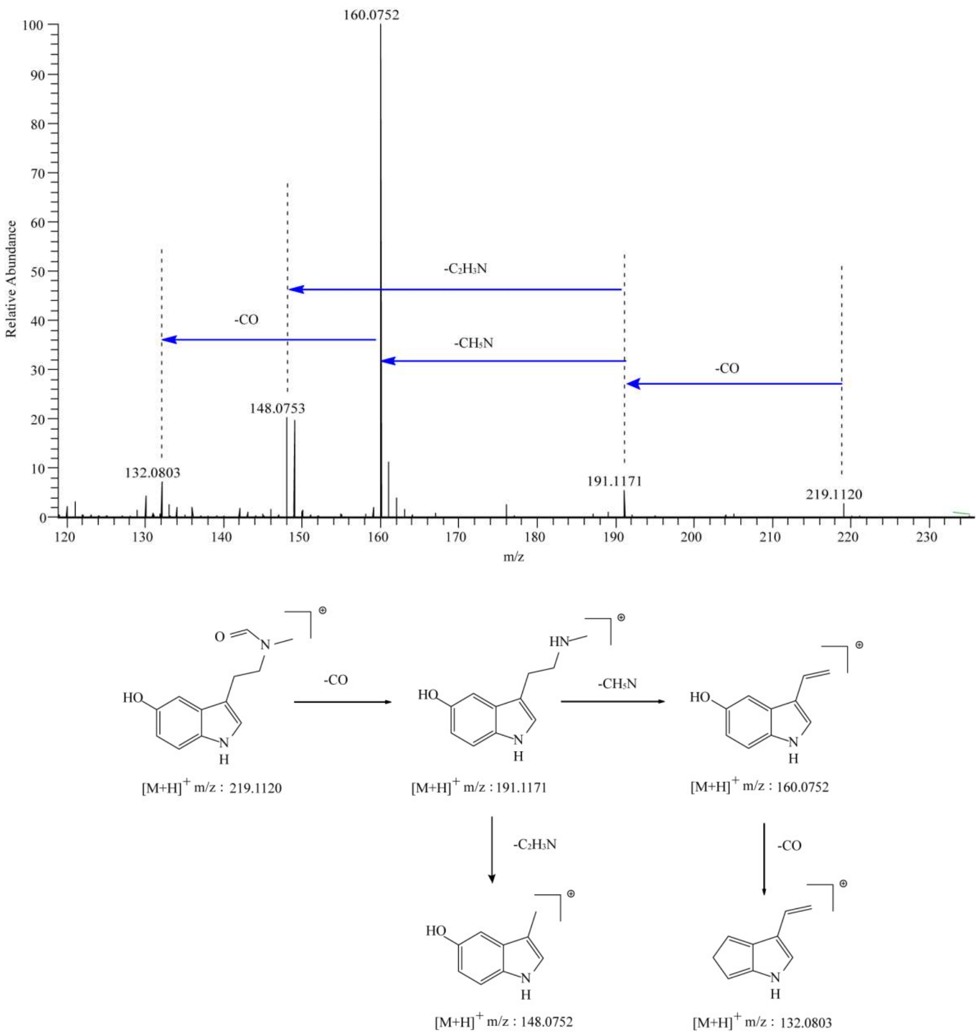

Figure S5. The MS/MS spectrum and fragmentation patterns of peak **20**

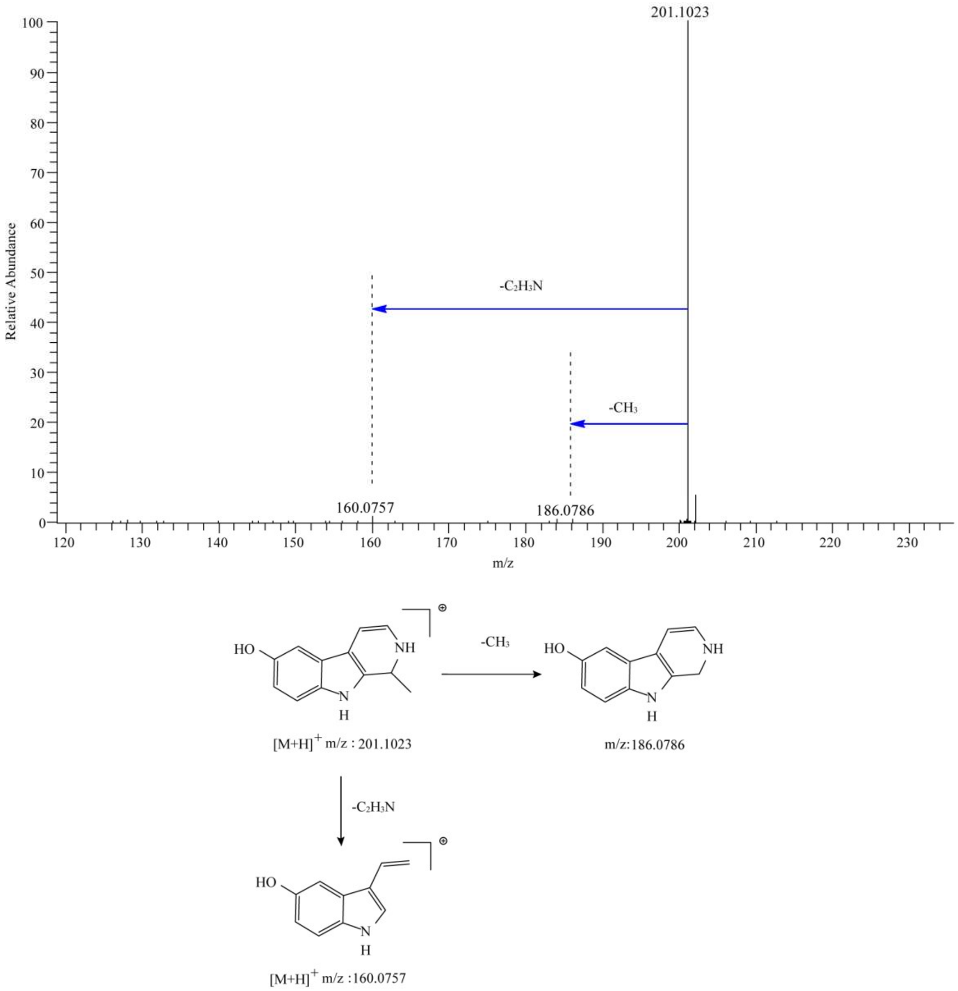

Figure S6 The MS/MS spectrum and fragmentation patterns of peak **31**
